# Early Microstructural Changes in the Left Inferior Fronto‐Occipital Fasciculus: A Key Factor in Probable Alzheimer's Disease Patients With Slow Gait Speed

**DOI:** 10.1002/brb3.71298

**Published:** 2026-03-12

**Authors:** Shan‐Wen Liu, Lin‐Lin Yao, Fang‐Bo Li, Xiao‐Ting Ma, Xiao‐Fen Weng, Meng Li, Jiangtao Zhu, Chun‐Feng Liu, Chun‐Yuan Zhang, Hua Hu

**Affiliations:** ^1^ Department of Neurology The Second Affiliated Hospital of Soochow University Suzhou China; ^2^ Department of Geriatric Medicine The Affiliated Suzhou Hospital of Nanjing Medical University, Suzhou Municipal Hospital Suzhou China; ^3^ Department of Imaging the Second Affiliated Hospital of Soochow University Suzhou China; ^4^ Department of Rehabilitation the Second Affiliated Hospital of Soochow University Suzhou China

**Keywords:** 6‐m gait speed, Alzheimer disease, cognition function, fixel‐based analysis, white matter

## Abstract

**Objective:**

To examine the association between alterations in specific fiber tracts and 6‐m gait speed and cognitive function in patients with clinically diagnosed Alzheimer's disease (AD) using fixel‐based analysis (FBA).

**Methods:**

Participants included 26 probable AD patients with slow gait speed (AD‐SGS), 28 without slow gait speed (AD‐nSGS), and 28 normal controls (NC).  All subjects underwent neuropsychological assessment, 6‐m gait speed testing, and cranial magnetic resonance imaging (MRI) scans. FBA was employed to derive white matter (WM) fiber metrics, including fiber density (FD), fiber cross‐sectional area (FC), and fiber density and cross‐sectional area (FDC). After adjustment for age and sex, the mean index values of the fiber tracts showing significant between‐group differences were extracted for the correlation between fiber properties, neuropsychological test scores, and parameters related to physical function.

**Results:**

AD‐SGS group exhibited significantly reduced FD and FDC in the left inferior fronto‐occipital fasciculus (IFOF_L) compared to AD‐nSGS group. In probable AD patients, FDC values of IFOF_L were positively correlated with global cognitive function, attention and calculation abilities, executive and visuospatial functions, and information processing speed. Furthermore, FD value of IFOF_L was positively correlated with 6‐m gait speed.

**Conclusions:**

In this clinically diagnosed AD patient with slow gait speed, the microstructural integrity of IFOF_L is closely linked to both cognitive decline and motor deficits. The structural deterioration of this tract is associated with the interplay between gait disturbance and impairments in executive and visuospatial functions, and information processing speed in this clinical context.

## Graphical abstract

Using fixel‐based analysis in Alzheimer's disease, we found that reduced fiber density value of the left inferior fronto‐occipital fasciculus is associated with both slower gait speed and impairments in executive and visuospatial functions, suggesting this white matter tract as a shared neural substrate for cognitive‐motor decline.

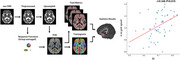

## 1 Introduction

Alzheimer's disease (AD), the most prevalent dementia worldwide, is characterized by progressive cognitive function, neuropsychiatric symptoms, and functional decline in activities of daily living. Currently, there are no disease‐modifying therapies that can effectively halt or reverse the progression of AD. Therefore, early detection and intervention are paramount to enable future treatments before irreversible neuronal damage occurs.

Emerging evidence indicates that gait slowing and balance deficits manifest early in AD, during the prodromal mild cognitive impairment (MCI) phase, and even at the preclinical stage (Hoogendijk et al. 2020; Rodriguez‐Molinero et al. 2019). Across all the stages of AD, alterations in body composition and reduced muscle strength are commonly observed, contributing to diminished physical function (Ogawa et al. 2018). This link is formalized by the concept of motoric cognitive risk syndrome (MCR), which identifies older adults with concurrent slow gait and subjective cognitive complaints in the absence of dementia or overt mobility disability (Meiner et al. 2020). A meta‐analysis of 17 studies (*n* = 1,206,782) established MCR as a robust predictor, significantly increasing the risk of cognitive impairment and dementia (RR = 2.20; 95%CI = 1.91–2.53) (Lim et al. 2024). Supporting its predictive value, a longitudinal study between 2000 and 2015 found that declining gait speed can precede measurable cognitive symptoms by up to 9 years and correlates with subsequent cerebrospinal fluid (CSF) amyloid‐β (Aβ) positivity, underscoring that motor slowing may reflect early AD neuropathology (Skillback et al. 2022).

Slowing gait speed in cognitive impairment is associated not only with global cognitive decline but also with deficits in specific domains—most prominently executive function, as well as attention, memory, verbal fluency, and visuospatial abilities (Umegaki et al. 2022; Toots et al. 2019). This connection implicates the integration of higher‐order cognition and motor planning in gait control, which relies on distributed neural circuits (Tieland et al. 2018). Supporting this, cohort studies link slower gait to reduced gray matter volume (GMV) in motor and cognitive‐related regions, such as the prefrontal cortex, motor areas, and cerebellum (Blumen et al. 2019), a pattern also observed in APOE‐E4 carriers with concomitant gait and cognitive deficits (Sakurai et al. 2024). Although AD is traditionally considered a gray matter disorder, emerging evidence underscores the early involvement of white matter (WM) pathology in the preclinical phase, as defined by NIA‐AA criteria (Depp et al. 2023; Jack et al. 2018). WM microstructural abnormalities can disrupt neural communication (Xu et al. 2024) and contribute to functional deficits prior to clinical symptoms (Bellaver et al. 2023). Animal models further indicate that axonal and myelin damage may even precede Aβ plaque deposition and neurofibrillary tangle formation (Chu et al. 2017). Thus, given their essential role in brain connectivity, WM tracts represent a critical target for further investigation in early AD (Pietroboni et al. 2020).

Chronic WM injury disrupts structural connectivity and neural signaling, while also exacerbating molecular pathologies such as oxidative stress and protein aggregation, thereby contributing to gait slowing in AD (Nasrabady et al. 2018; Lindh‐Rengifo et al. 2023). Consistent with this, individuals with AD exhibit more severe periventricular white matter hyperintensities (PWMH) in frontal‐parietal regions compared to MCI and normal controls, with this WMH burden correlating with specific gait impairments, including reduced velocity and altered swing time (Ogama et al. 2021). However, the mechanistic role of specific WM alterations in mediating the relationship between cognitive decline and motor deterioration remains unexplored, a gap that this study aims to address.

Current research on WM microstructure largely relies on diffusion tensor imaging (DTI). However, DTI suffers from a fundamental limitation: its inability to resolve complex fiber configurations such as crossing fibers, which occur in up to 90% of WM voxels at conventional resolutions  (Winston 2012; Jeurissen et al. 2013). When fibers within a voxel show opposing changes—for instance, one set with reduced integrity and another with enhanced connectivity—DTI cannot distinguish them. Consequently, while voxel‐averaged metrics like fractional anisotropy may indicate regional abnormalities, they lack specificity to individual fiber populations and are ambiguous to interpret. This issue is particularly pronounced in AD, where WM pathology involves multifaceted processes—including axonal loss, demyelination, and gliosis—occurring across microscopic and macroscopic scales. These concurrent changes further confound traditional DTI analyses. An emerging technique, fixel‐based analysis (FBA), overcomes these limitations by modeling diffusion data at the level of individual fiber populations within each voxel (Dhollander et al. 2021). A “fixel” refers to a specific fiber bundle within a voxel with a distinct orientation, enabling separate quantification of micro‐ and macrostructural properties per population. FBA derives three principal metrics:  (1) fiber density (FD), reflecting intra‐axonal volume and microstructural integrity; (2) fiber cross‐section (FC), a macrostructural index of bundle cross‐sectional area; and (3) fiber density and cross‐section (FDC), a combined measure of overall pathway integrity sensitive to neurodegeneration (Mito et al. 2018). By avoiding the spatial averaging inherent in DTI, FBA offers enhanced specificity in identifying fiber‐specific alterations. Its growing application in AD research has provided novel insights into WM degeneration with improved pathological specificity (Dewenter et al. 2023; Lebrun et al. 2024).

Utilizing the FBA framework, this study aims to map the micro‐ and macro‐structural alterations of WM tracts onto the interplay between gait and cognitive deficits in patients with probable AD. We propose that the deterioration of specific WM pathways constitutes a shared neural substrate underlying the co‐occurrence of these clinical features. Our analysis seeks to clarify this structural link, ultimately informing strategies to preserve motor function and potentially mitigate disease progression.

## 2 Materials and Methods

### 2.1 Participants

A total of 54 patients with probable AD were recruited from the Memory Disorders Clinic at the Department of Neurology, the Second Affiliated Hospital of Soochow University, between June 2022 and June 2024. The study protocol was approved by the Institutional Review Board of the hospital (Approval No.: JD‐LK2023031‐I01), and written informed consent was obtained from all participants and their caregivers prior to enrollment. Additionally, 28 normal controls (NC) exhibiting normal cognitive function and gait speed were included. Inclusion criteria for AD patients were as follows: ① diagnosis based on the core clinical criteria established by the NIA–AA workgroups in 2011, either at present assessment or previously confirmed (McKhann et al. 2011); ② age between 55 and 90 years and right‐handedness; ③ absence of space‐occupying lesions or significant periventricular and deep WM lesions inconsistent with age as assessed by cranial magnetic resonance imaging (MRI) (Fazekas et al. 1987); ④ Hachinski Ischemia Score ≤ 4; ⑤ no use of medications affecting cognitive function or mental status within 1 month prior to enrollment; and ⑥ completion of clinical assessments and ability to comprehend the required neuropsychological scales. Exclusion criteria for patients with probable AD comprised: ① other disorders contributing to cognitive impairment, including cerebrovascular disease and intracranial tumors; ② significant systemic medical diseases, such as cardiac, pulmonary, hepatic, or renal dysfunction, hypothyroidism, malignancy, or other chronic wasting illnesses; ③ major psychiatric disorders including severe depression or schizophrenia; ④ contraindications to bioelectrical impedance analysis or MRI due to metallic implants; ⑤ musculoskeletal or balance impairments affecting limb mobility, such as lumbar disc herniation, previous fractures, diabetic foot, or osteoarthritis; ⑥ presence of motor symptoms including subjective fatigue, muscle weakness, atrophy, or signs of pyramidal or extrapyramidal tract involvement; and ⑦ a Mini Nutritional Assessment score ≤ 24 (Liu et al. 2022).

NC participants were enrolled according to the following criteria: ① aged 55 to 90 years and right‐handed; ② a score of ≥26 points on the Montreal Cognitive Assessment (MoCA). ③ absence of WM abnormalities on MRI and no history of central nervous system disorders; ④ free of any psychoactive medications for at least 1 month prior to enrollment; ⑤ gait speed exceeding 1 m/s. The same exclusion criteria applied to both the probable AD and NC cohorts.

### 2.2 Demographic and Clinical Data

Sex, age, years of education, hypertension, diabetes, hyperlipidemia, coronary heart disease, smoking history and alcohol consumption, height, and weight were systematically documented. Body Mass Index (BMI) was derived as weight in kilograms divided by the square of height in meters. Low physical activity was defined as walking less than 120 min per week for women and less than 150 min per week for men (Jia et al. 2019).

### 2.3 Parameters Related to Physical Function and Body Composition Analysis

① 6‐m gait speed was assessed over a 6‐m distance at usual walking pace using a stopwatch. The average of three trials was recorded. A slowed gait speed was defined as ≤1.0 m/s (Chen et al. 2020). Based on this measure, patients with probable AD were categorized into those with slow gait speed (AD‐SGS, *n* = 26) and those without (AD‐nSGS, *n* = 28). ② Hand grip strength was evaluated as a marker of physical function using a dynamometer (WCS‐100, Nantong, China). Participants performed three maximal squeezes with each hand, and the maximum value across all trials was recorded (Chen et al. 2020). ③ The 5‐times sit‐to‐stand (5‐STS) time was administered to assess functional lower limb strength. Participants were instructed to stand fully and sit back down five times as quickly as possible from a standardized armless chair (height: 46 cm), with arms crossed over the chest. The total time to complete the task was recorded. ④ Appendicular skeletal muscle mass index (ASMI) was derived from bioelectrical impedance analysis (BIA; Inbody 720, Biospace Ltd., Seoul, Korea). ASMI was calculated as the sum of skeletal muscle masses in all four limbs divided by height squared (kg/m^2^).

### 2.4 Neuropsychological Assessments

All neuropsychological assessments were conducted by trained assessors who had undergone standardized protocol training to ensure consistency. Testing was performed in a quiet environment and encompassed both cognitive and non‐cognitive domains. Cognitive function was evaluated using the following instruments: the MoCA for global cognition; the Clinical Dementia Rating (CDR) scale, which assesses the overall cognitive function, including memory, orientation, judgment, social and occupational functioning, domestic and recreational activities, and self‐care and personal hygiene; the Clock Drawing Test (CDT) for visuospatial and executive functions; the Memory and Executive Screening scale (MES), yielding a total score (MES‐T) with subscores for memory (MES‐M) and executive function (MES‐E); the Digit Symbol Substitution Test (DSST) for processing speed; the Digital Span Test (DST), including both forward (FDST) and backward (BDST) conditions to assess attention and working memory, respectively; serial subtraction tasks (e.g., subtracting 7 serially from 100) to evaluate attention and mental calculation; and the Verbal Fluency Test (VFT) for verbal function. Non‐cognitive tests include the Activities of Daily Living (ADL) scale to evaluate functional independence and the 17‐item Hamilton Depression Rating Scale (HAMD‐17) to quantify the severity of depressive symptom.

### 2.5 WMH Evaluation

For the evaluation of WMH, brain MRI T2 fluid‐attenuated inversion recovery (FLAIR) images were obtained using the following equipment: 3.0T (Prisma) MRI scanner (TR = 7000 ms; TE = 135 ms, TI = 2000 ms, field of vision = 256 mm × 256 mm, slice thickness = 4 mm, interslice gap = 1 mm). Based on the images, WMHs were categorized into grades 0–3 according to the Fazekas classification (Chen et al. 2020). Deep white matter hyperintensity (DWMH) and periventricular hyperintensity (PWMH) were each graded on a 4‐point scale (0–3). DWMH severity was defined as follows: 0, absent; 1, punctate foci (< 3 mm maximum diameter); 2, beginning confluence of foci (≥ 3 mm); and 3, large confluent areas. PWMH was graded as: 0, absent; 1, cap or pencil‐thin lining; 2, smooth halo; and 3, irregular PWMH extending into deep WM. Two neurologists independently performed the grading; discrepancies were resolved by consensus to assign the final grade.

### 2.6 Magnetic Resonance Imaging (MRI) Acquisition and Preprocessing

**FIGURE 1:**
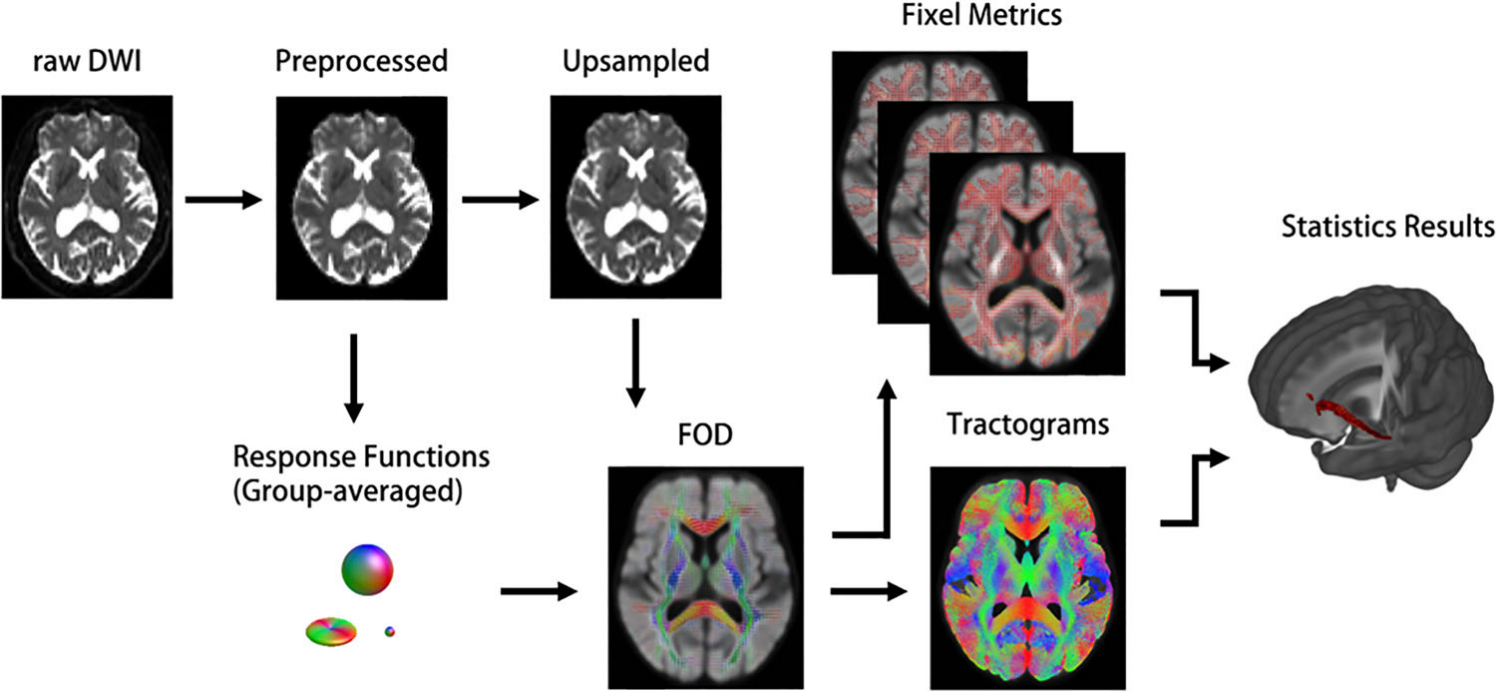
Flowchart of diffusion MRI processing and the process based on FBA.

The diffusion MRI processing and analysis in this study followed a standardized full process (see Figure 1: Flowchart of diffusion MRI processing and the process based on FBA). Diffusion‐weighted imaging (DWI) was performed on a 3.0T Siemens Prisma MRI scanner (Germany) using the following acquisition parameters: 60 diffusion‐weighted volumes with *b* = 2000 s/mm^2^ and 5 non‐diffusion‐weighted volumes (*b* = 0 s/mm^2^); repetition time/echo time = 6600/86 ms; isotropic voxel size = 2.2 mm^3^; phase encoding direction = anterior‐to‐posterior (AP). Total acquisition time for the DWI sequence was approximately 9 min. Preprocessing and subsequent FBA were conducted using an established pipeline, as described previously (Dhollander et al., 2021). We used dcm2niix to convert DICOM files to NIfTI files. All dMRI data were preprocessed using the mrtrix (https://www.mrtrix.org) tool. The preprocessing steps included: ① denoising; ② gibbs ringing correction; ③use mrtrix dwifslpreproc command called FSL head dynamic eddy current correction (https://fsl.fmrib.ox.ac.uk/fsl/fslwiki), and adjusted according to the dynamic eddy current correction of image transformation BVector; ④ ANTs was used for N4 bias field correction, which was used to correct the uneven low‐frequency intensity in MRI image data; ⑤ resampling the DWI data to isotropic resolution of 1.25 mm^3^ to provide a standard spatial reference for subsequent calculations (Figure 1: Upsampled). The preprocessing quality of all the dispersed data was quantitatively evaluated, and the key quality control indicators showed no systematic differences among the three groups (see Tables S1, S2, and S3).

### 2.7 Fixel‐Based Analysis

After these preconditioning steps, the response functions for WM, GM, and CSF were calculated for all subjects, and group‐average response functions were subsequently obtained. Using the toolkit MRtrix3Tissue (https://3Tissue.github.io), combined with the Single‐Shell 3‐Tissue Constrained Spherical Deconvolution algorithm, WM fiber orientation distributions (FOD) were calculated (Figure 1: Response Functions → FOD). A group‐level FOD template (Figure 1: Group‐averaged) was created based on the FOD maps of 28 NC subjects, which was used for subsequent spatial standardization and analysis. The FOD of all subjects was registered to the FOD template, and FD, FC, and FDC were calculated. Since FC is usually skewed, log_FC (logarithm) is calculated and used in subsequent analyses, rather than FC, to ensure a normal distribution of variables. The total number of fiber bundles was 20 million, and then 2 million fiber bundles were filtered by SIFT (Spherical deconvolution Informed Filtering) method.

### 2.8 Fixel‐based Metrics

FBA was employed to derive FD and FC at the fixel level (Raffelt et al., 2017). A “fixel” refers to a specific fiber population within a single voxel, enabling distinct FD and FC measurements even in regions with complex fiber crossings. ① FD is computed from FOD maps, as the integral of the FOD along a given orientation correlates with the intra‐axonal volume aligned in that direction. Consequently, FD serves as a sensitive marker of microstructural alterations within the voxel. ② FC reflects macrostructural changes: axonal loss leads to a reduction in the cross‐sectional area of a fiber bundle, indicating decreased spatial occupancy of that tract (Raffelt et al. 2017). By leveraging nonlinear spatial normalization from subject to template space, structural variations can be quantified for each FC in the plane orthogonal to the fixel direction. ③ FDC, the multiplicative combination of FD and FC, captures concomitant micro‐ and macrostructural pathological changes, thereby enhancing sensitivity to the overall capacity of a fiber population to support communication between cortical regions.

### 2.9 Statistical Analysis

All statistical analyses were performed using the SPSS software package (version 26.0). Continuous variables following a normal distribution are presented as means ± standard deviation (SD) and were compared across groups using one‐way Analysis of Variance (ANOVA), with the F statistic reported. Where ANOVA indicated significant differences, post‐hoc comparisons were conducted with Bonferroni adjustment for multiple testing. Non‐normally distributed continuous variables are summarized as medians and interquartile range, and were analyzed using the Kruskal–Wallis test (reported as H statistic). Categorical variables are expressed as percentages (%) and compared using Pearson's χ^2^ test or Fisher's exact test, as appropriate, with the χ^2^ value indicated. The Bonferroni method was applied to correct for multiple comparisons where applicable. A two‐sided *p* value of less than 0.05 was considered statistically significant.

### 2.10 Whole‐brain Fixel‐Based Analysis

To localize regions exhibiting alterations in FD, FC, and FDC among the AD‐SGS, AD‐nSGS and NC groups, we initially conducted whole‐brain FBA. For inference, we employed a non‐parametric permutation‐based framework combined with connectivity‐based fixel enhancement (CFE) to account for multiple comparisons across the fixel‐wise network. This approach provides strong control over family‐wise error (FWE) rates. For the whole‐brain FBA, group comparisons were performed using general linear models (GLMs) adjusted for age and sex. This standardized model served as an exploratory analysis to identify the most significant WM alterations associated with differences in 6‐m gait speed among the clinical groups. These analyses specifically aimed to assess the association's robustness, particularly against vascular factors, by further adjusting for global cognition and WMH in subsequent models. Statistical differences in FBA‐derived metrics across the three groups were assessed via ANOVA. We ran 5000 permutations to generate null distributions for each contrast (Nichols and Holmes 2002), assigning FWE‐corrected *P*‐values to all fixels. Effects were considered significant at a threshold of *p* < 0.05 after FWE correction.

For post‐hoc analysis following significant ANOVA results, mean FD, log‐FC, and FDC values were extracted across all participants. Statistical maps were thresholded using MRtrix3 tools, and fixed masks were generated from significant clusters identified in the ANOVA. These masks were subsequently used to extract subject‐level average values for each metric (Figure 1: Statistics Results). The final statistical maps were registered to the Montreal Neurological Institute (MNI) standard space for anatomical labeling, using the deformation field derived from warping the group‐averaged FOD to MNI space. We then examined correlations between FD and FDC values within significant fiber tracts and measures of neuropsychological performance, physical function, and body composition, while controlling for age, sex, and years of education. These analyses specifically aimed to assess the association's robustness, particularly against vascular factors, by further adjusting for global cognition and WMH in subsequent models. All reported correlations were assessed using non‐parametric permutation testing to compute family‐wise error (FWE) corrected *p* values.

## 3 Results

### 3.1 Comparison of General Clinical Characteristics Among the Three Groups

As demonstrated in Table 1, the NC group included 12 males and 16 females, aged (69.79 ± 8.30) years with 11.0 (9.0,16.0) years of education. The AD‐nSGS group included 7 males and 21 female patients, aged (70.39 ± 9.09) years with 8.5 (2.5,12.0) years of education. The AD‐SGS group included 6 males and 20 female patients, age (73.81 ± 8.71) years with 8.0 (0.0,12.0) years of education. The height, weight, ASMI, 6‐m gait speed, 5‐STS time, grip strength, and MTA scores were statistically significant differences (all *p *< 0.05). Compared to the NC and AD‐nSGS groups, the AD‐SGS group had lower ASMI, slower 6‐m gait speed, longer 5‐STS time, poorer grip strength, and higher MTA scores (all *p *< 0.01).

**TABLE 1:** Comparison of general clinical characteristics among the three groups.

Index	NC (*n* = 28)	AD‐nSGS (*n* = 28)	AD‐SGS (*n* = 26)	*F/H/χ*2	*p*
Male, *n* (*%*)	12 (42.9)	7 (25.0)	6 (23.1)	3.093^c^	0.213
Age (*y*)	69.79 ± 8.30	70.39 ± 9.09	73.81 ± 8.71	1.654^a^	0.198
**Years of education (*y*)**	11.0 (9.0,16.0)	8.5 (2.5,12.0)	8.0 (0.0,12.0)^e^	6.949^b^	**0.031** ^*^
Hypertension, *n* (*%*)	11 (39.3)	15 (53.6)	14 (53.8)	1.535^c^	0.464
Diabetes, *n* (*%*)	5 (17.9)	2 (7.1)	8 (30.8)	5.04^c^	0.08
Dyslipidemia, *n* (%)	5 (17.9)	7 (25.0)	7 (26.9)	0.702 ^c^	0.704
Coronary artery disease, *n* (%)	3 (10.7)	4 (14.3)	6 (23.1)	1.564 ^c^	0.479
Current smoking, *n* (*%*)	4(14.3)	2(7.1)	3 (11.5)	0.817^c^	0.756
Alcohol drinking, *n* (*%*)	3 (10.7)	4 (14.3)	2 (7.7)	0.657^c^	0.905
**Height (cm)**	160.56 ± 8.44	157.14 ± 7.02	153.49 ± 8.58^e^	5.386^a^	**0.006** ^**^
**Weight (kg)**	63.34 ± 10.12	58.83 ± 9.93	54.55 ± 9.46^e^	5.248^a^	**0.007** ^**^
BMI (kg/m^2^)	24.51 ± 3.13	23.80 ± 3.53	23.07 ± 2.93	1.369^a^	0.260
Low physical activity, *n* (*%*)	7 (25.0)	10 (35.7)	12 (46.2)	2.64^c^	0.267
**ASMI (kg/m^2^)**	6.71 ± 0.98	6.45 ± 0.86	5.86 ± 1.05^e^, ^f^	5.519^a^	**0.006** ^*^ ^*^
**6‐m gait speed**	1.30(1.24,1.39)	1.21 (1.11,1.28)^d^	0.91 (0.76,0.96) ^e^, ^f^	58.832^b^	**0.000** ^*^ ^*^
**5‐STS time**	9.77 (8.61,11.36)	11.07 (9.59,12.16)	12.48 (10.76,15.80) ^e^	17.082^b^	**0.000** ^*^ ^*^
**Grip strength (kg)**	27.55(21.75,31.4)	23.80 (17.80,31.0)	17.85 (16.10,25.1) ^e^	12.447^b^	**0.002** ^**^
**MTA**	1.0 (0.0,1.0)	2.0 (1.0,2.0)^d^	2.0 (2.0,3.0)^e^, ^f^	19.037^b^	**0.000** ^**^
**PWMH**	1.0 (0.0,1.0)	1.0 (0.0,1.0)	1.0 (0.0,1.0)	0.002^b^	**0.999**
**DWMH**	1.0 (1.0,1.0)	1.0 (1.0,1.0)	1.0 (1.0,1.0)	0.114^b^	**0.944**

Abbreviations: 5‐STS, five‐times sit‐to‐stand test; AD‐LGS, probable AD patients with slow gait speed; AD‐nSGS, probable AD patients without slow gait speed; ASMI, appendicular skeletal muscle mass index; BMI, body mass index; MTA, medial temporal atrophy; NC, normal controls. PWMH, periventricular white matter hyperintensity; DWMH, Deep white matter hyperintensity

^a^ Normally distributed variable is expressed as mean ± standard deviation and analyzed by ANOVA of three groups (*F* value).

^b^ Non‐normally distributed variable is expressed as median (interquartile range) and analyzed by Kruskal–Wallis *H* test of three groups (*H* value).

^c^ Categorical variables are expressed as frequency (percent; *χ* ^2^ value).

^d^ AD‐nSGS significantly different from NC (*p* < 0.05);

^e^ AD‐SGS significantly different from NC (*p* < 0.05);

^f^ AD‐SGS significantly different from AD‐nSGS (*p* < 0.05).

* *p *< 0.05; ^**^ *p *< 0.01.

### 3.2 Comparison of Neuropsychological Characteristics Among the Three Groups

As demonstrated in Table 2, compared with the NC group, the probable AD group had lower scores for MoCA, CDT, MES‐T, MES‐M, MES‐E, DSST, DST, FDST, BDST, VFT, and higher scores for ADL, HAMD‐17, and CDR (all *p* < 0.05); compared with the AD‐nSGS group, the AD‐SGS group had lower scores for CDT, MES‐T, MES‐E, DSST, DST, BDST, and calculation, and higher scores for CDR (*p* < 0.05).

**TABLE 2:** Comparison of neuropsychological characteristics among the three groups.

Scale	NC (*n* = 28)	AD‐nSGS (*n* = 28)	AD‐SGS (*n* = 26)	*p* Value	
				NC vs. AD	AD‐nSGS vs. AD‐SGS
MoCA	26.9 ± 1.3	15.8 ± 4.2	13.4 ± 5.9	0.000^**^	0.091
**CDR**	0.0 (0.0,0.0)	0.75 (0.5,1.0)	1.0 (1.0,2.0)	0.000^**^	0.007^**^
CDT	3.0 (3.0,3.0)	2.0 (1.5,2.0)	2.0 (1.0,2.0)	0.000^**^	0.232
**MES‐T**	88.9 ± 5.9	57.8 ± 14.0	47.4 ± 14.8	0.000^**^	0.010^*^
MES‐M	41.0 (39.0,45.0)	20.5 (16.0,22.5)	19.5 (15.0,24.0)	0.000^**^	0.924
**MES‐E**	47.0 ± 3.8	37.0 ± 9.3	28.4 ± 10.7	0.000^**^	0.003^**^
**DSST**	29.7 ± 8.4	15.9 ± 9.1	11.4 ± 6.7	0.000^**^	0.041^*^
**DST**	13.0 (12.0,14.0)	12.0 (11.0,13.0)	10.0 (9.0,13.0)	0.000^**^	0.027^*^
FDST	8.0 (8.0,9.0)	8.0 (7.5,8.5)	8.0 (6.0,9.0)	0.035^*^	0.344
**BDST**	5.0 (4.0,5.0)	4.0 (3.0,5.0)	3.0 (2.0,4.0)	0.000^**^	0.004^**^
**Calculation**	5.0 (5.0,5.0)	5.0 (2.5,5.0)	2.5 (1.0,4.0)	0.000^**^	0.004^**^
VFT	33.6 ± 10.8	18.1 ± 5.7	15.5 ± 7.1	0.000^**^	0.141
ADL	20.0 (20.0,20.5)	24.0 (23.0,25.5)	25.0 (23.0,31.0)	0.000^**^	0.351
HAMD‐17	3.0 (0.5,4.5)	5.0 (2.0,6.0)	6.0 (4.0,7.0)	0.000^**^	0.228

Abbreviations: AD, Alzheimer's disease; AD‐nSGS, probable AD patients without slow gait speed; AD‐SGS, probable AD patients with slow gait speed; ADL, Activities of Daily Living scale; BDST, Backward Digit Span Test; CDR, clinical dementia rating; CDT, Clock Drawing Test; DSST, Digit Symbol Substitution Test; DST, Digit Span Test; FDST, Forward Digit Span Test; HAMD‐17, 17‐item Hamilton Depression Rating Scale; MES, Memory and Executive Screening scale; MES‐E, Executive Factor Score of MES; MES‐M, Memory Factor Score of MES; MES‐T, total score of MES; MoCA, Montreal Cognitive Assessment; NC, normal controls; VFT, Verbal Fluency Test.

*p < 0.05; **p<0.01.

### 3.3 Comparison of FD, FC, and FDC in TOI Among the Three Groups

**FIGURE 2:**
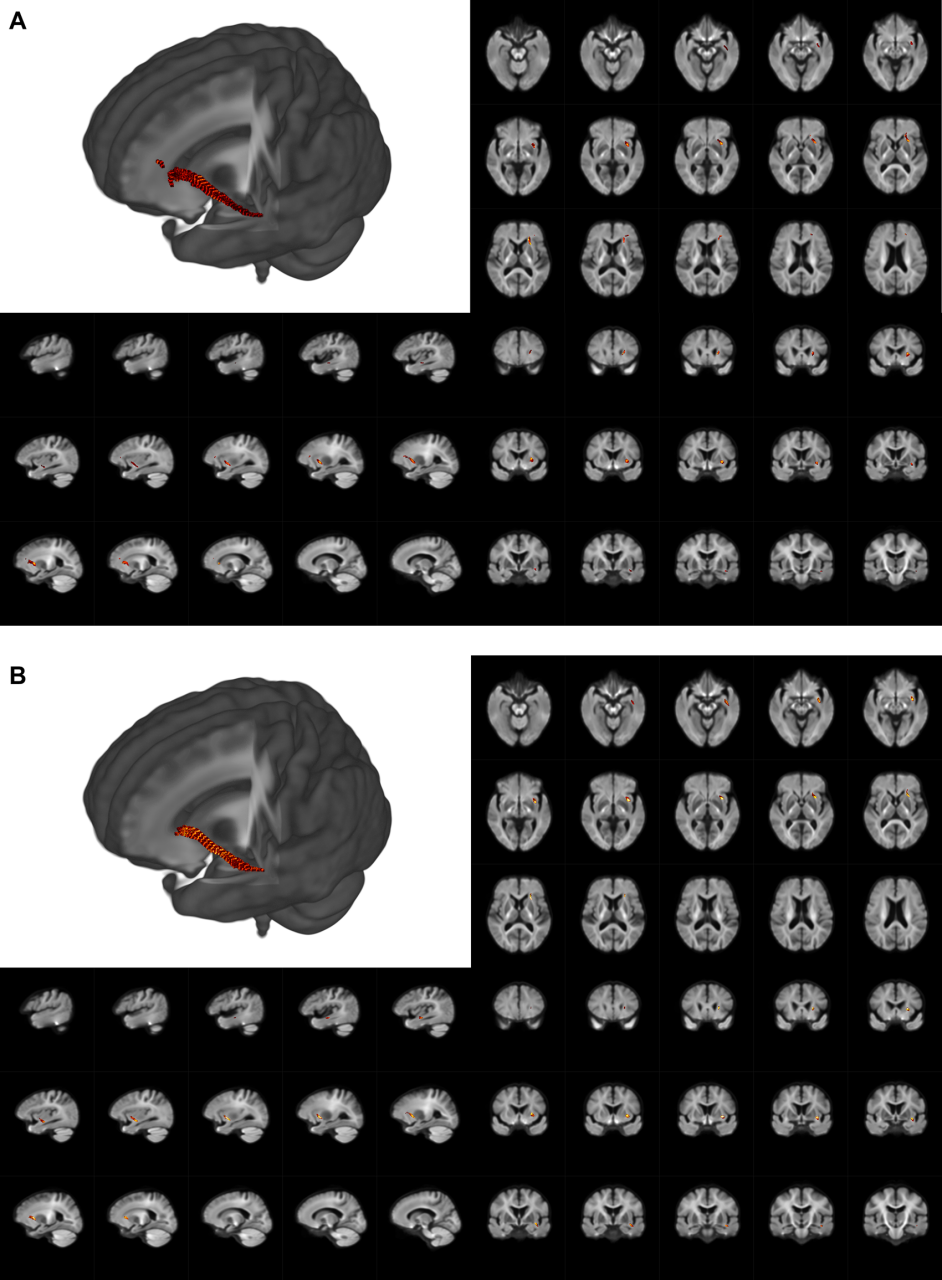
Whole‐brain FBA results. Fiber tract‐specific differences among AD‐nSGS, AD‐SGS, and NC are shown. Streamline segments were cropped from the template tractogram to include only those corresponding to fixels significant at family‐wise error‐corrected(FWE) *p *< 0.05. (A) Fiber tract‐specific difference between groups of FD (3D view, axial view, sagittal view, coronal view); (B) Fiber tract‐specific difference between groups of FDC (3D view, axial section view, sagittal section view, coronal section view).

As demonstrated in Figure 2, Tables 3, and 4, adjusting for age and sex, streamline segments associated with fixels that had a significant (FWE‐corrected *p* value < 0.05) decrease between the three groups in FD and FDC of the left inferior fronto‐occipital fasciculus (IFOF_L); there were no significant differences among the three groups in FC.

**TABLE 3:** Regions with intergroup differences in the FD index.

Region	MNI coordinate			*F* value	Cohen‘s *f*	Number of fixel
	*X*	*Y*	*Z*			
Left inferior fronto‐occipital fasciculus	−21	28	1	14.7	0.618	271

*Note*: Effect sizes were calculated as Cohen‘s f for ANOVA; *f* ≥ 0.4 denotes a large effect.

Abbreviation: MNI, Montreal Neurological Institute.

**TABLE 4:** Regions with intergroup differences in the FDC index.

Region	MNI coordinate			*F* value	Cohen‘s *f*	Number of fixel
	*X*	*Y*	*Z*			
Left inferior fronto‐occipital fasciculus	−33	6	8	10.7	0.527	251

*Note*: Effect sizes were calculated as Cohen‘s *f* for ANOVA; *f* ≥ 0.4 denotes a large effect.

Abbreviation: MNI, Montreal Neurological Institute.

### 3.4 Two‐by‐Two Post Hoc Comparisons of FD and FDC of the Statistically Different IFOF_L Among the Three Groups

**FIGURE 3:**
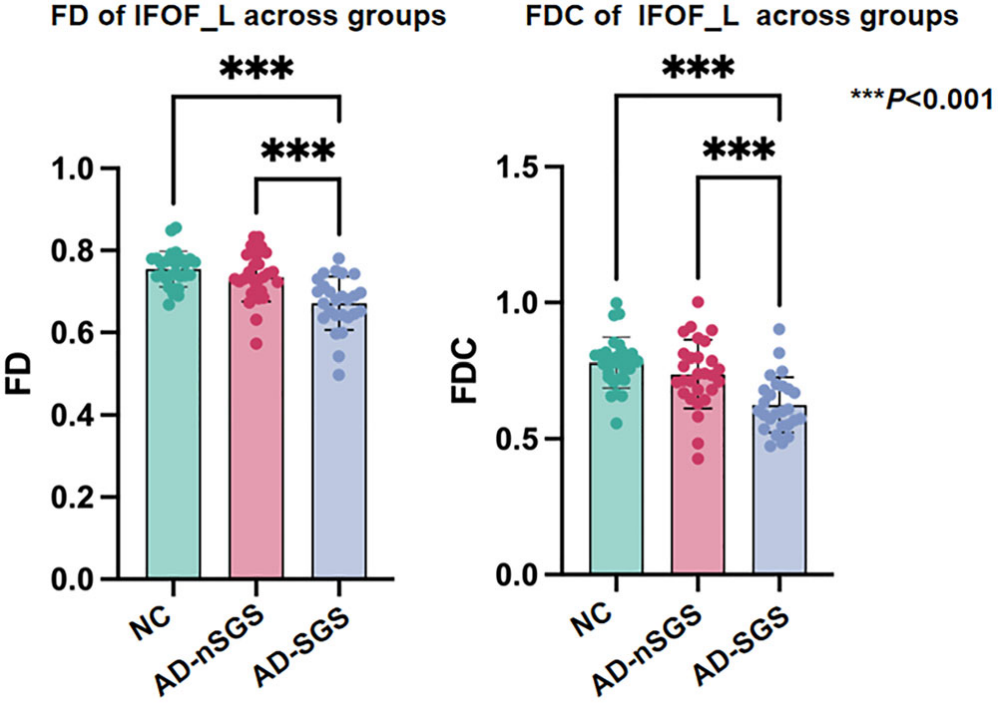
Results of post‐hoc pair‐wise comparisons of mean FD values and FDC values extracted from the significant fixels after ANOVA analysis. IFOF_L, left inferior fronto‐occipital fasciculus.

As demonstrated in Figure 3, post hoc test analysis showed that compared to the AD‐nSGS group and NC group, FD and FDC in the AD‐SGS group were significantly lower in the IFOF_L.

### 3.5 Correlation Between FDC and MoCA, CDT, MES‐T, MES‐E, DSST, DST, and Calculation Scores of Cerebral IFOF_L With Statistical Differences Between Groups

**FIGURE 4:**
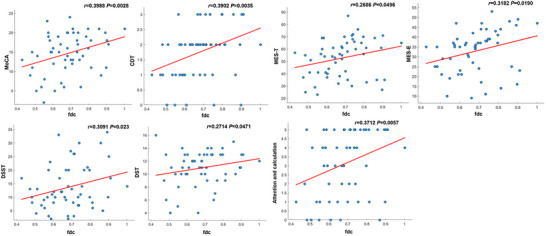
Correlations between extracted values of mean FDC in the IFOF_L of probable AD patients and neuropsychological scales.

As demonstrated in Table 5 and Figure 4, correlation analysis showed that after controlling for age, sex, and years of education, the scores of MoCA (*r* = 0.40, *p* = 0.003), CDT (*r* = 0.39, *p* = 0.004), MES‐T(*r* = 0.27, *p* = 0.0496), MES‐E (*r* = 0.32, *p* = 0.02), DSST (*r* = 0.31, *p* = 0.02), DST (*r* = 0.27, *p* = 0.047), and calculation (*r* = 0.37, *p* = 0.006) were positively correlated with the FDC values of probable AD patients in the IFOF_L (all *p *< 0.05).

**TABLE 5:** Correlation between FD and FDC in left inferior fronto‐occipital fasciculus that were statistically different between groups with neuropsychological scales.

	FD		FDC	
IFOF_L	*r*	*p* Value	*R*	*p* Value
**MoCA**	0.2799	**0.0404**	0.3988	**0.0028**
CDR	−0.0605	0.6641	−0.1996	0.1479
**CDT**	0.3316	**0.0143**	0.3902	**0.0035**
**MES‐T**	0.2828	**0.0382**	0.2686	**0.0496**
MES‐M	0.0549	0.6935	0.0815	0.5582
**MES‐E**	0.3572	**0.0080**	0.3182	**0.0190**
**DSST**	0.2863	**0.0358**	0.3091	**0.0230**
**DST**	0.1669	0.2276	0.2714	**0.0471**
FDST	0.0609	0.6620	0.2372	0.0842
BDST	0.2353	0.0868	0.24397	0.0755
**Attention and calculation**	0.3451	**0.0106**	0.3712	**0.0057**
VFT	0.2428	0.0769	0.1880	0.1733
ADL	0.0146	0.9168	−0.0873	0.5302
HAMD‐17	−0.2108	0.1261	−0.2684	0.0497

*Note*: The values present *r* (*p* value);*r*, correlation coefficient; correlation analysis adjusted for age, sex, and education. Bold values denote statistical significance (*p* < 0.05).

Abbreviations: ADL, Activities of Daily Living scale; BDST, Backward Digital Span Test; CDR, Clinical dementia rating; CDT, Clock Drawing Test; DSST, Digit Symbol Substitution Test; DST, Digital Span Test; FD, fiber density; FDST, Forward Digital Span Test; FDC, combined fiber density and cross‐section; HAMD‐17, the 17‐item Hamilton Depression Scale‐17; IFOF_L, left inferior fronto‐occipital fasciculus; MES, Memory and Executive Screening scale; MES‐E, Executive Factor Score of MES; MES‐M, Memory Factor Score of MES; MES‐T, Total Score of MES; MoCA, Montreal Cognitive Assessment; VFT, Verbal Fluency Task.

## 4 Correlation Between FDC and 6‐m Gait Speed and 5‐STS Time of Cerebral IFOF_L With Statistical Differences Between Groups

**FIGURE 5:**
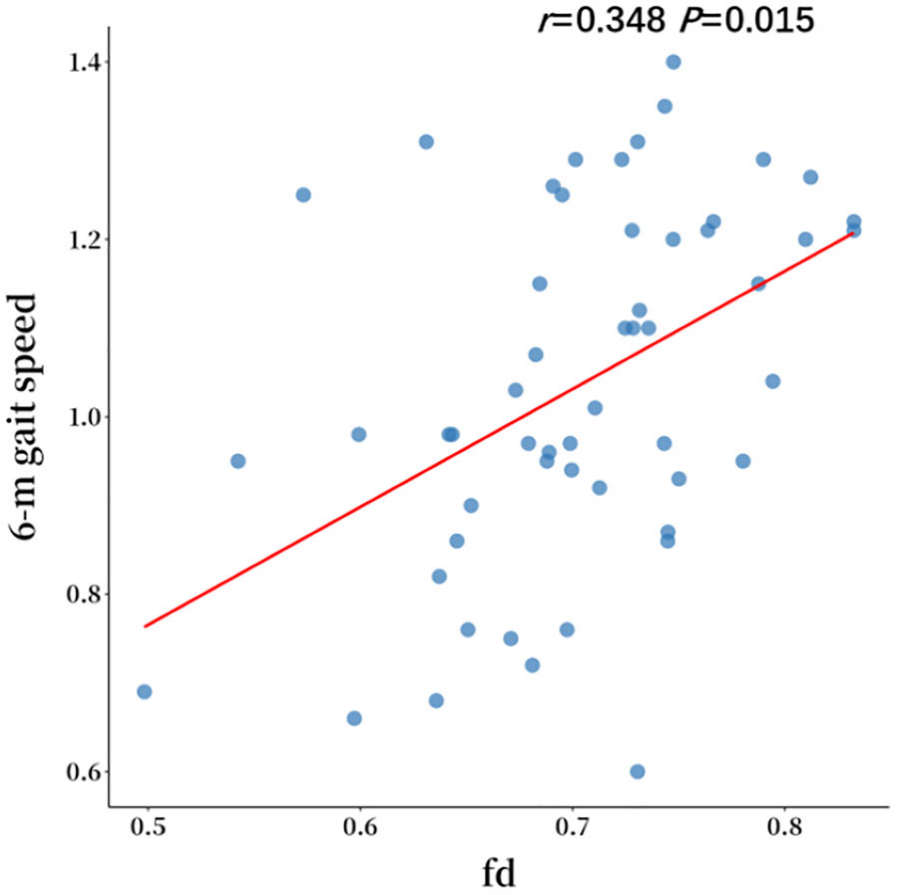
Correlations between extracted values of mean FD in the IFOF_L of probable AD patients.

To complement the group analysis and address the continuous relationship between 6‐m gait speed and WM integrity, we performed a sensitivity analysis using 6‐m gait speed as a continuous variable across all probable AD patients. As demonstrated in Table 6 and Figure 5, correlation analysis showed that after controlling for age, sex, years of education, MoCA, PWMH, and DWMH, 6‐m gait speed (*r* = 0.348, *p* = 0.015) was positively correlated with the FD values of probable AD patients in the IFOF_L (*p *< 0.05).

**TABLE 6:** Correlation between FD and FDC in left inferior fronto‐occipital fasciculus with parameters related to physical function and body composition analysis.

	FD		FDC	
IFOF_L	*r*	*p* value	*r*	*p* value
ASMI	0.05	0.733	0.039	0.794
**6‐m gait speed**	0.348	**0.015**	0.272	0.061
5‐STS time	−0.18	0.220	−0.161	0.275
Grip strength	0.259	0.076	0.233	0.111

*Note*: The values present *r* (*p* value);*r*, correlation coefficient; correlation analysis adjusted for age, sex, and education. Bold values denote statistical significance (*p* < 0.05).

Abbreviations: 5‐STS, 5‐times sit‐to‐stand; ASMI, appendicular skeletal muscle mass index; FD, fiber density; FDC, combined fiber density and cross‐section; IFOF_L, left inferior fronto‐occipital fasciculus.

## 5 Discussion

The results of this study indicate that, compared to the AD‐nSGS and NC groups, the AD‐SGS group showed reduced micro‐structural integrity in the IFOF_L. Compared to the NC group, the AD‐SGS group showed a more extensive cognitive decline. When compared to the AD‐nSGS group, the AD‐SGS group scored lower on the CDR, MES‐T, MES‐E, DSST, DST, BDST, and calculation, indicating worse performance in overall cognitive function, attention and calculation, executive ability, and information processing speed. Additionally, the FDC values of the IFOF_L in probable AD patients were correlated with MoCA, CDT, MES‐T, MES‐E, DSST, DST, and calculation scores, and the FD values of the IFOF_L were correlated with 6‐m gait speed, suggesting an association between the integrity of this tract and both cognitive performance and gait speed in this clinically diagnosed AD cohort. These findings support the micro‐structural changes in the IFOF_L are associated with both gait slowing and multi‐domain cognitive decline in clinically diagnosed AD patients. Microstructural changes in WM structure and function in probable AD patients should be closely monitored, and proactive enhancement of gait speed or somatic function through training may be beneficial in preventing and delaying the onset and progression of probable AD.

Slow gait speed has been identified as an early indicator of cognitive decline and an elevated risk of dementia in later life (Collyer et al. 2022). Furthermore, a progressive reduction in walking pace correlates with impairments across multiple cognitive domains (Lu et al. 2024). The interrelationship among frailty, cognition, and gait is complex and non‐coincidental, likely reflecting underlying impairments in shared neural circuits. These circuits are vulnerable to age‐related neurodegeneration, microvascular pathology, and diffuse WM damage (Montero‐Odasso and Hachinski 2014).  Normal gait control relies on the integrated function of sensory processing, frontal executive operations, motor planning, attention, and cognitive oversight. Consistent with this, macrostructural brain alterations are frequently observed in individuals with MCI and AD, such as reductions in GMV and degradation of WM architecture (Zhong et al. 2023). Additionally, compromised integrity of WM tracts has been linked to postural instability and slowed gait speed among community‐dwelling elderly individuals (He et al. 2021). These studies suggest that abnormalities in WM may represent one of the mechanisms underlying cognitive decline and slow gait speed. WM disconnection can interfere with cortical function, and several studies have established a strong link between decreased gait speed and cortical volume. For instance, community‐dwelling participants with slower gait speeds show not only reduced total GMV, but also GMV loss in the frontal and parietal lobes (Lee et al. 2019). We hypothesize that WM, serving as critical fiber pathways connecting distributed cortical areas, plays a critical role in facilitating efficient information transfer and supporting integrated cognitive and motor functions. AD is a syndrome characterized by progressive brain network dysfunction, typified by amyloid‐β deposition, neurofibrillary tangulation, and spatially patterned cortical atrophy accompanied by loss of functional connectivity (Mito et al. 2018). Throughout this process, WM is essential for maintaining normal communication between cortical regions. In early AD, microstructural WM alterations initially emerge within the cingulum bundle, subsequently progressing to short‐range association fibers linking temporal and parietal cortices, and ultimately affecting long‐range projection pathways to the frontal lobe (Teipel et al. 2016). These compromised WM tracts contribute to degradation within functional networks highly vulnerable to AD pathology, such as the default mode network, thereby underpinning broad cognitive deterioration (Yang et al. 2023). Consequently, the characteristic WM structural changes in AD patients likely disrupt the function of related cortical regions, associated with cognitive decline and reduced gait speed. It is noteworthy that the median 6‐m gait speed of our AD‐nSGS group was 1.21 m/s, which is slower than expected for healthy older adults. This observation underscores the pervasive nature of motor involvement in AD, suggesting that subtle gait slowing may be a common feature of the disease spectrum. Our use of the ≤1.0 m/s cutoff (Chen et al. 2020), which aligns with the Asian Working Group for Sarcopenia (AWGS) 2019 consensus criteria for “possible sarcopenia”, was intended to identify a distinct subgroup with marked mobility impairment within the probable AD population. The fact that we still detected significant differences in WM integrity between the AD‐SGS and AD‐nSGS groups, despite the latter also showing some slowing, likely indicates that our reported effect sizes are conservative estimates. Indeed, the true neuroanatomical differences associated with clinically significant gait impairment in AD might be even more pronounced. In line with this, our findings demonstrate that patients in the AD‐SGS group exhibited specific microstructural alterations in the IFOF_L tract alongside widespread cognitive impairment, compared to both the NC and AD‐nSGS groups. This converging evidence suggests that changes in specific WM pathways, such as the IFOF_L, may represent a shared neural correlate of both cognitive decline and reduced gait speed in probable AD patients. However, it must be emphasized that the tract‐specific effects observed in the IFOF_L cannot be attributed uniquely to Alzheimer's pathology. In the absence of biomarker confirmation, the microstructural alterations reported here may equally stem from or be co‐influenced by cerebrovascular disease or other age‐related neurodegenerative processes commonly present in clinical dementia populations.

Several studies based on structural imaging and DTI have examined the correlations between WM micro‐structural differences and gait speed in healthy and pathological cognitive aging. For instance, Snir et al. (2019) examined WM tract regions, including the corpus callosum, forceps major and minor, longitudinal fasciculus, fronto‐occipital fasciculus, and the corticospinal tract. They found that among individuals with MCI, DTI metrics—namely, fractional anisotropy, mean diffusivity, axial diffusivity, and radial diffusivity—were associated with slower gait speed. These parameters reflect variations in water diffusion characteristics and microstructural tissue integrity. Consistent with these findings, other investigations have reported similar correlations between structural MRI or DTI metrics and gait stability (Bruijn et al. 2014), frailty (Tian et al. 2020; Ducca et al. 2023), and physical activity (Boa Sorte Silva et al. 2023; Tian et al. 2022). While these studies offer compelling evidence that loss of WM integrity is associated with impaired gait performance in adults, especially those with neurodegenerative diseases, they are constrained by a fundamental methodological limitation: conventional structural MRI and DTI cannot definitively distinguish whether observed abnormalities arise from axonal loss or dysmyelination. While DTI enables visualization of macroscopic WM tracts, it lacks fiber‐specific resolution and struggles to accurately model diffusion in regions with complex crossing fibers, which constitute up to 90% of cerebral WM voxels. This shortcoming is especially critical in neurodegenerative pathologies, where coexisting micro‐ and macrostructural alterations further complicate WM architecture and undermine the interpretability of diffusion‐derived metrics (Jeurissen et al. 2013; Li et al. 2020). Notably, the FBA framework enables tract‐specific statistical comparisons by leveraging constrained spherical deconvolution—a higher‐order diffusion model capable of resolving multiple fiber orientations within a single voxel (Tournier et al. 2008). Within this framework, quantitative metrics are derived from “fixels”, corresponding to individual fiber populations per voxel. These quantitative metrics include FD reflecting microscopic changes in intra‐axonal volume, FC, a marker of macroscopic alterations in a cross‐sectional area of WM tracts, and FDC, a composite measure derived from their product. Notably, empirical evidence indicates that a decrease in axonal density or volume may not immediately reduce FC if the extracellular space becomes occupied by glial proliferation, inflammatory infiltrates, or extracellular matrix. Subsequent clearance of these components, however, can lead to tract atrophy and a decline in FC, suggesting that reductions in FD often precede macroscopic atrophy (Dhollander et al. 2021). This supports the view that FD is more sensitive to early neurodegenerative processes, while FC decline reflects later‐stage structural degradation. By integrating both microscopic and macroscopic aspects, the combined metric FDC offers enhanced sensitivity to incipient neurodegeneration across entire fiber tracts. This is one of the reasons why FDC, along with FD and FC, was primarily used in our study for intergroup comparisons and correlation analyses, capitalizing on their complementary pathological specificity and highlighting a strength of our research. Thus, utilizing FBA techniques, our study identified a significant reduction in the FD and FDC of the IFOF_L in the AD‐SGS group compared to the AD‐nSGS and NC groups. This finding suggests that the microstructural integrity of the IFOF_L is associated with the process of gait slowing in this clinical AD cohort.

This study also found that AD‐SGS patients showed significant axonal damage on the microstructure of the IFOF_L, which may be one of the sensitive nerve fiber bundles associated with slow gait speed in probable AD patients. This observation aligns with findings reported by Snir et al. (2019), who reported that MCI patients presenting both dual‐task gait deficits and frailty exhibit preferential microstructural alterations in WM tracts supporting executive and visuospatial processing, such as the IFOF_L, corpus callosum, and small forceps. In addition, we found that after adjusting for age, sex, education, MoCA, PWMH, and DWMH, the FD value of the IFOF_L of probable AD patients was still correlated with 6‐m gait speed, and after adjusting for age, sex, education, the FDC value was also positively correlated with MoCA, CDT, MES‐T, MES‐E, DSST, DST, and calculation scores. These findings are consistent with the anatomical premise that the IFOF_L, as a component of fronto‐occipital networks involved in executive processing, shows structural alterations that correlate with slowed gait (Montero‐Odasso et al. 2014). Microstructural compromise of this fronto‐occipital tract, reflected by reduced fixel‐based metrics such as FD and FDC, may relate to a disruption of functional integration between distributed cognitive and sensory regions. Specifically, such degradation could disrupt communication linking prefrontal executive circuits with visuospatial and proprioceptive processing areas located in occipital and parietal cortices (Genova et al. 2013). This disruption appears to initially affect the left hemisphere, which is consistent with previous evidence suggesting that left‐sided WM damage is a cause of executive dysfunction. It is necessary to clearly state that the inter‐group comparison model of whole‐brain FBA in this study (adjusting for age and gender) is an exploratory framework used to identify the most significant WM difference patterns related to the decline in 6‐m gait speed. The results represent “phenotype‐related overall WM changes”. Our subsequent sensitivity analysis indicates that the composite index (FDC) of the IFOF_L is no longer significantly associated with 6‐m gait speed after controlling for the burden of WMH. This finding is crucial as it suggests that in the clinical possible AD cohort, the correlation between FDC and 6‐m gait speed may simultaneously reflect the combined effects of neurodegeneration and vascular pathology. In contrast, the association between the FD of the IFOF_L and 6‐m gait speed remains robust after controlling for WMH, suggesting that the axonal‐level microdamage of this pathway has a more direct association with the decline in gait speed, independent of cerebral vascular lesions.

IFOF is one of the longest WM tracts in the ventral aspect of the human brain, extending from anterior to posterior regions. It sends fibers connecting the parieto‐occipital cortex, the basal temporal lobe, the insula, and the superior parietal lobule to the frontal lobe, and traverses structures, such as the external capsule and the extreme capsule (Gonzalez Alam et al. 2024), providing an important anatomical basis for brain function. Furthermore, the IFOF naturally intersects with several other major tracts, such as the superior longitudinal fasciculus, arcuate fascicle, inferior longitudinal fasciculus, and middle longitudinal fascicle (Sarubbo et al. 2013). Extensive evidence supports a strong association between the microstructural integrity of IFOF and multiple cognitive domains, including memory, reading, attention, and visual processing (Wu et al. 2016, Tian et al. 2016). Anatomically, the IFOF comprises dorsal and ventral subdivisions, each potentially subserving distinct functional roles (Roux et al. 2021). Martino et al. (2010) identified the superficial dorsal subcomponent of the IFOF connects superior occipital regions involved in visually guided behavior with posterior superior temporal and parietal regions, ultimately projecting to the posterior inferior frontal gyrus. This dorsal pathway is well positioned to support integrated visual − spatial processing and action control. By contrast, the deep ventral subdivision links occipitotemporal and ventromedial temporal regions, which are implicated in object recognition, to various frontal areas, including nodes within the anterior and ventral default mode network and dorsolateral prefrontal regions associated with cognitive control. This anatomical arrangement suggests a role for the ventral IFOF in facilitating visually guided decision‐making and semantic control. These findings align with the observed correlations between the IFOF_L and executive function, visuospatial processing, and information integration capacity demonstrated in our study. Moreover, functional MRI studies have consistently demonstrated that frontal and parietal regions form an integrated circuit—the fronto‐parietal control network—which underpins executive function and cognitive control (Lo et al. 2017). Conversely, disruption of WM tracts connecting these regions has been associated with declines in both motor performance and executive abilities in patients with MCI or AD (Yang et al. 2021). This may also explain the association between the IFOF, which connects the fronto‐parietal WM pathway, and slower gait speed. Hence, our findings suggest that damage to the IFOF_L microstructures, which are involved in sensory integration, motor and executive processes, and cognitive control, may demonstrate a stronger association with slowed gait speed in probable AD patients than in other brain regions.

## 6 Conclusion

This study found that, compared to NC and AD‐nSGS patients, AD‐SGS patients exhibited the earliest micro‐structural integrity damage in the IFOF_L, along with a more extensive cognitive decline. AD‐SGS patients performed worse than AD‐nSGS patients in overall cognitive function, attention and calculation, executive abilities, and information processing speed. Microstructural alterations in the IFOF_L, which are linked to deficits in specific cognitive domains, are also associated with the presence of gait slowing and somatic function decline in this clinically diagnosed AD cohort. Gait slowing may be a manifestation of both WM micro‐structural changes and multi‐domain cognitive impairments in the brains of this clinically diagnosed AD cohort. These findings expand our understanding of the pathway linking WM tract structural changes, gait slowing, and cognitive decline. Further studies with larger cohorts are needed to validate these findings and to investigate the relationship between these changes and the underlying pathophysiology. Recognizing the role of WM structural or functional changes in the cognitive decline and gait slowing of probable AD patients, targeted interventions aimed at modifying WM structure or function, along with training to enhance somatic function, may be beneficial in delaying the progression of probable AD. This approach has potential clinical significance for effective screening, diagnosis, and treatment.

This study has several limitations: (1) Owing to its cross‐sectional design, causal inferences between WM integrity, gait speed, and cognitive decline cannot be established. Therefore, longitudinal designs are needed to elucidate these temporal relationships. (2) The sample size is relatively small, particularly for male participants, and no gender stratification was performed. Expanding cohorts to include additional individuals with MCI and probable AD would improve generalizability. Furthermore, we adopted a strict FWE correction to control the risk of Type I errors in whole‐brain multiple comparisons. However, this conservative correction strategy, combined with our relatively small sample size, may have reduced statistical power and increased the risk of Type II errors. Therefore, the significant WM fiber tracts identified in this study may be relatively robust; while potential effects that did not reach the strict threshold need to be replicated in future larger independent samples. Furthermore, although we adopted a strict spatial standardization process, the relatively small sample size may affect the reproducibility and accuracy of the statistical maps at the fine anatomical boundaries. Therefore, the currently identified significant clusters should be regarded as an ‘interesting region’, and the precise range and the distinction from adjacent fiber bundles need to be further confirmed in larger‐scale samples. (3) Future investigations should incorporate more granular gait metrics, such as stride length, coefficient of variation, and stride time variability (Beauchet et al. 2018), and attempt both single‐task and dual‐task gait tests to better capture motor‐cognitive interactions (de Oliveira Silva et al. 2020). (4) While single b‐value acquisitions can yield robust fixel‐based metrics (Jeurissen et al. 2013), the use of multi‐shell diffusion MRI with higher *b *values would enhance anatomical accuracy in modeling complex fiber configurations.  Subsequent studies would benefit from adopting such advanced imaging protocols to strengthen methodological rigor. (5) AD diagnoses were clinically derived and lack biomarker confirmation. As such, while the cohort represents a typical clinical AD population, it may include individuals with mixed neuropathologies. This heterogeneity complicates the definitive attribution of observed microstructural damage in the IFOF_L specifically to Alzheimer's pathology, rather than to vascular or other age‐related co‐pathologies. This potential misclassification could influence our results in two principal ways. First, a dilution effect may have led to underestimation of the true effect sizes, as the inclusion of non‐AD cases could attenuate associations specifically driven by AD pathology. Second, given that IFOF_L integrity is vulnerable to both AD and vascular pathologies—and that slow gait is also linked to vascular health—the association observed between IFOF_L integrity and gait speed may partly reflect shared vascular pathophysiology. Notwithstanding these limitations, all participants underwent comprehensive clinical and neuroimaging assessments to exclude other major causes of dementia, ensuring the cohort's relevance to real‐world clinical practice. The strong association identified between this specific tract and coexisting cognitive and motor deficits remains clinically meaningful. Incorporating biomarker‐defined AD cohorts (e.g., using Aβ‐PET or CSF biomarkers) will be essential to disentangle the distinct contributions of Alzheimer's versus vascular pathology to the gait‐cognitive interplay described here in our future studies. (6) We did not include WMH as a covariate in the main model and acknowledge that gait speed is a complex phenotype influenced by multiple factors (including cerebrovascular health). Future research needs to simultaneously evaluate the independent and interactive effects of AD pathology, cerebrovascular disease, and other factors on gait speed in a larger sample.

## Author Contributions

Study concept and design: Hua Hu, Chun‐yuan Zhang, and Chun‐feng Liu. Acquisition of data: Shan‐wen Liu, Meng Li, Xiao‐fen Weng, and Jiangtao Zhu. Analysis and interpretation of data: Shan‐wen Liu, Lin‐Lin Yao, and Fang‐bo Li. Drafting of the manuscript: Shan‐wen Liu and Xiao‐ting Ma. Critical revision of the manuscript for important intellectual content: Hua Hu, Chun‐yuan Zhang, and Chun‐Feng Liu. Software, formal analysis, visualization, writing – original draft, review, and editing: Shan‐wen Liu. Investigation, data curation, and validation: Lin‐Lin Yao. Software, data curation, and validation: Fang‐Bo Li. Investigation and methodology: Xiao‐Ting Ma. Formal analysis and conceptualization: Xiao‐fen Weng. Formal analysis and conceptualization: Meng Li. Software and methodology: Jiangtao Zhu. Supervision and project administration: Chun‐Feng Liu. Validation, supervision, and project administration: Chun‐yuan Zhang. writing – review and editing, conceptualization, supervision, project administration: Hua Hu. All authors reviewed the manuscript.

## Funding

This work was supported by the National Natural Science Foundation of China (62475179); Jiangsu Provincial Medical Key Discipline (ZDXK202217); Suzhou Applied Basic Research (Medical health) Science and Technology Innovation Project—Youth project (SYW2024081); Research Project of Neurological Diseases in the Second Affiliated Hospital of Soochow University Research Center (ND2023A01, ND2024A01); Pre‐research Fund Project for Clinical Application at the Second Affiliated Hospital of Soochow University (SDFEYLC2343); Brain Health Youth Fund—Precision Diagnosis and Treatment of Alzheimer's Disease Research in 2024; Suzhou Medical College‐QiLu Medical Research Program of Soochow University (24QL200210).

## Ethics

The study was performed in accordance with the guidelines of the 1964 Declaration of Helsinki and was approved by the ethics committee of the Second Affiliated Hospital of Soochow University (JD‐LK2023031‐I01). All patients provided written informed consent prior to the study.

## Conflicts of Interest

The authors have no conflicts of interest.

## Supporting information




**Supplementary Table**: brb371298‐sup‐0001‐TableS1.csv

**Supplementary Table**: brb371298‐sup‐0002‐TableS2.csv

**Supplementary Table**: brb371298‐sup‐0003‐TableS3.csv

## Data Availability

The datasets analyzed during the current study are available from the corresponding author on reasonable request.
